# Effects of sleep deprivation on food-related Pavlovian-instrumental transfer: a randomized crossover experiment

**DOI:** 10.1038/s41598-024-60223-2

**Published:** 2024-05-01

**Authors:** Wai Sze Chan

**Affiliations:** Room 664, 6/F, Department of Psychology, The Jockey Club Tower, Centennial Campus, The University of Hong Kong, Pokfulam Road, Hong Kong, Hong Kong SAR

**Keywords:** Learning and memory, Motivation, Reward, Psychology, Human behaviour

## Abstract

Recent research suggests that insufficient sleep elevates the risk of obesity. Although the mechanisms underlying the relationship between insufficient sleep and obesity are not fully understood, preliminary evidence suggests that insufficient sleep may intensify habitual control of behavior, leading to greater cue-elicited food-seeking behavior that is insensitive to satiation. The present study tested this hypothesis using a within-individual, randomized, crossover experiment. Ninety-six adults underwent a one-night normal sleep duration (NSD) condition and a one-night total sleep deprivation (TSD) condition. They also completed the Pavlovian-instrumental transfer paradigm in which their instrumental responses for food in the presence and absence of conditioned cues were recorded. The sleep × cue × satiation interaction was significant, indicating that the enhancing effect of conditioned cues on food-seeking responses significantly differed across sleep × satiation conditions. However, this effect was observed in NSD but not TSD, and it disappeared after satiation. This finding contradicted the hypothesis but aligned with previous literature on the effect of sleep disruption on appetitive conditioning in animals—sleep disruption following learning impaired the expression of appetitive behavior. The present finding is the first evidence for the role of sleep in Pavlovian-instrumental transfer effects. Future research is needed to further disentangle how sleep influences motivational mechanisms underlying eating.

## Introduction

Sleep curtailment is a prevalent problem across age groups and populations around the world, and is accompanied by serious public health consequences^1^. The National Sleep Foundation recommends that adults should obtain seven to nine hours of sleep per night^2^; however, population-based studies have found that 20–30% of adults report getting fewer than six hours of sleep per night^3,4^. Similarly, obesity is a public health crisis in developed countries, with obesity rates also rising at alarming rates in developing countries over the past two decades^5^. Previous research suggests a potential link between these two pressing public health issues—insufficient sleep elevates the risk of obesity^6–8^, with metabolic, hormonal, and behavioral mechanisms proposed to account for the association^9^.

One of the plausible mechanisms underlying the relationship between sleep deprivation and obesity is that sleep deprivation alters the way we eat. Indeed, experimentally induced partial or full sleep deprivation has been found to increase hunger, caloric consumption, and neurological responsiveness to food rewards in humans^10–14^. A meta-analysis showed that, on average, experimental sleep deprivation increased caloric consumption by 385 calories per day in humans^15^. Although sleep loss appears to affect eating, the underlying processes remain unclear. Some previous studies have found that sleep loss alters metabolic and hormonal mechanisms in humans, such as up-regulating ghrelin, the hormone signaling hunger, and down-regulating leptin, the hormone signaling satiety^16,17^; however, mixed findings have been observed in later studies^18^. A recent partial sleep deprivation experiment in adolescents has found that sleep loss affects caloric consumption only in the evening during the extended period of wakefulness^19^, suggesting that sleep loss may increase caloric consumption merely because of the increased opportunities to eat. Previous studies have shown that the impact of sleep loss on eating in humans is stronger when food rewards are hyperpalatable, i.e., calorie-dense, high-fat, and high-carbohydrate, with the enhanced impact not fully explained by increased hunger^20–22^. These findings suggest that sleep deprivation may affect both homeostatic eating and hedonic eating; the former is dependent on hunger, and the latter is driven by the palatability of food with or without the presence of homeostatic needs^23^.

There are times when we eat for neither hunger nor pleasure. For instance, “I can’t resist eating everything on my plate even when I feel full and satiated” and “I find myself munching on snacks whenever I am watching television” exemplify *habitual control of eating*. Habitual control of behavior is defined as cue-elicited processes, learned via repeated associations, and is insensitive to outcome devaluation^24^. Although habitual control of eating is closely related to hedonic eating, the wanting of a food (i.e., a motivation to eat a food) and the liking of a food (i.e., the affective reactivity to a food) are well-differentiated constructs in appetite research with dissociable neurological substrates^25^. Although it is widely believed to influence the way we eat, habitual control of eating has been considered the culprit in problematic eating only recently^26^. As habitual control of eating is characterized by insensitivity to outcome devaluation, it is linked to overeating (eating despite the decreased value of food after satiation) and external eating (eating in response to environmental cues rather than hunger), both of which are frequently associated with obesity in observational research^27^. Indeed, individuals with a higher body mass index (BMI) were found to less sensitive to food reward devaluation than those with a lower BMI^28^.

Habitual control and goal-directed control are two parallel processes that compete or interact to direct behavior^24,29,30^. Although it may not be adaptive in the pursuit of goals, habitual control is more efficient than goal-directed control and may compete better or function more adaptively in circumstances in which cognitive resources are depleted^31^. Deficits in executive functioning following sleep loss are well-documented^32^. As such, sleep loss may tip the balance between habitual and goal-directed control in favor of more efficient, less effortful habitual control at times when cognitive resources are depleted following sleep loss. Indeed, observational research has shown that individuals who regain weight after weight loss have a shorter sleep duration than those who maintain weight loss^33^. It is possible that insufficient sleep might increase the likelihood of resorting to old eating and lifestyle habits, an indication of overreliance on habitual control of behavior. An experimental study has shown that one-night total sleep deprivation (TSD) biased responding to stimuli associated with the devalued outcome in the slips-of-action task, which the authors interpreted as overreliance on habitual control of behavior over goal-directed control^34^. However, the greater biased responding for a devalued outcome documented by Chen et al.^34^ could also be explained by the confounding impact of sleep loss on information encoding^35^. Furthermore, greater responsiveness to a devalued outcome in the slips-of-action task could also be attributable to excessive goal-directed behavior rather than an overreliance on habitual control of behavior.

An increased desire to eat when one is exposed to food-associated cues is a widely observed process even in normal eaters^36^, whereas a continued desire to eat triggered by cues despite satiation is more strongly implicated in overeating and disordered eating^27^. The Pavlovian-instrumental transfer (PIT) paradigm can be used to dissociate these two processes and allows for the evaluation of the effects of sleep deprivation on each of these processes. The PIT is an experimental paradigm for examining the influence of conditioned stimuli (CS) on instrumental behavior^37^ and has been used to evaluate the effect of cues on food-seeking behavior^38–40^. It can be used in conjunction with satiation procedures to evaluate cue-elicited food-seeking behavior in the presence and the absence of satiation. The transfer effects derived from the PIT paradigm refer to the phenomenon where the presence of CS paired with an appetitive outcome (CS+) enhances an instrumental action. There are two types of transfer effects. Specific transfer refers to the increased instrumental responding in the presence of an outcome-specific CS that signals the outcome previously trained with the instrumental action via instrumental conditioning, i.e., response-outcome (R-O) learning. Increased responding can be expressed in the increased preference for the instrumental action (e.g., greater percentage of the choice of action against alternative actions) or the increased frequency of the action; sometimes it can also be expressed in suppressed responding for the alternative actions^37^. A real-life example would be an increased desire to order pizza after seeing a pizza commercial. General transfer refers to the increase in the vigor of an instrumental action in the presence of a general CS that signals an outcome that is not previously trained with the instrumental action but generates a similar motivational state, e.g., an approach action. For instance, an increase in approach behavior towards food in the presence of a CS associated with other food. A real-life example would be an increased desire to order pizza after seeing a non-pizza food commercial.

Taken together, habitual control of food-seeking behavior, defined as being cue-elicited and insensitive to satiation, could be a potential mechanism underlying the relationship between sleep deprivation and increased risks of obesity. However, no previous studies have examined the effects of sleep deprivation on these processes. Hence, the present study aimed to evaluate whether sleep deprivation had an impact on the effects of Pavlovian cues and satiation on food-seeking behavior, measured with the PIT paradigm, using a randomized, within-individual crossover experimental design in which participants partook in one-night TSD and one-night normal sleep duration (NSD). It was hypothesized that participants’ instrumental behavior would be more strongly affected by cues, i.e., greater specific and general transfer effects, and less sensitive to satiation following one-night TSD compared to NSD.

## Results

### Descriptive statistics

One-hundred-twenty-one adults who met the habitual sleep duration criterion confirmed by actigraphy enrolled in the experiment. Twenty-five of them dropped out before completing the experiment. Data collection ended after 96 participants had completed the experiment, as re-activated COVID-related social distancing measures led to a halt in further data collection. Table 1 presents the sample’s demographic and clinical characteristics. Eighteen participants pressed no keys during the PIT or failed to learn the response-outcome (R-O) or the stimulus-outcome (S-O) associations. Their data were thus excluded from the main analysis, leaving 78 participants. The excluded participants did not differ from those included in any of the demographic or clinical variables.

**Table 1:** Means and standard deviations of demographic and clinical variables (N = 78).

	Mean	SD	Range
Age (years)	25.68	8.44	18–57
Gender	50% men	–	–
Education level			
Secondary or below	11.5%	–	–
Bachelor level	62.5%	–	–
Masters and doctorates	26.9%	–	–
Body mass index	21.61	3.75	16.61–40.16
PSQI global scores	3.65	1.23	1–5
Sleep Duration	8.15	0.78	6–10
MEQ	50.94	6.07	42–72
DASS_depression	2.90	3.10	0–12
DASS_Anxiety	2.67	2.77	0–8
EDDS	6.31	4.25	0–16

*Note.* PSQI—Pittsburgh sleep quality index. MEQ—Morningness–eveningness questionnaire. DASS—depressive, anxiety, and stress scales. EDDS—eating disorder diagnostic scale.

Table 2 presents the differences in the measures of food liking, hunger, stress, total caloric consumption during satiation, working memory, response inhibition, and delay discounting between the TSD and NSD before and after satiation (see Fig. 5 in “Methods” for experimental procedures). None of the variables differed between them except for subjective liking of the hyperpalatable food and stress. Participants liked the hyperpalatable food less and felt more stressed in TSD than NSD. As expected, subjective liking of most food items, hunger, and stress were significantly reduced after satiation. Unexpectedly, despite selecting food items based on similar prior ratings of subjective liking (see “Methods” section “Selection of food rewards”), subjective liking of the two hyperpalatable food (O1 and O3) was significantly lower than that of the two non-hyperpalatable food (O2 and O4) after tasting (paired* t* = − 2.73, *p* = 0.008). It should be noted that a larger than expected portion of data were missing on food liking, hunger, and stress due to a programming error resulting in missing data on these variables in the first 20 participants. Sporadic missing data on other variables were caused by non-systematic data file corruption for two participants.

**Table 2:** Means and standard deviations of ratings of hunger, stress, and measures of covariates in TSD and NSD conditions (N = 78).

	*N*	NSD		TSD	
		Before satiation	After satiation	Before satiation	After satiation
O1 liking^a,b^	56	69.31 (23.67)	65.38 (26.96)	65.25 (26.49)	58.12 (28.27)
O2 liking^b^	56	76.34 (20.72)	70.90 (25.29)	73.38 (23.43)	70.26 (25.27)
O3 liking^b^	56	67.66 (24.30)	63.18 (26.97)	61.81 (28.17)	57.97 (28.39)
O4 liking	56	71.41 (23.05)	68.66 (24.40)	66.13 (27.90)	65.38 (27.72)
Hunger^b^	56	66.00 (20.01)	10.31 (12.61)	64.95 (25.73)	11.20 (14.52)
Stress^a,b^	56	23.65 (18.03)	14.50 (13.77)	36.83 (26.89)	22.12 (21.78)
3-back (%)	74	74.51 (17.47)		71.88 (20.45)	
Go no go	74	95.78 (9.28)		96.13 (3.77)	
Delay discounting	74	0.08 (0.16)		0.09 (0.13)	
Calorie consumed (kcal)	71	611 (267)		604 (265)	

*Note.* TSD—total sleep deprivation. NSD—normal sleep duration. O1—hyperpalatable food reward used in instrumental and Pavlovian training. O2—non-hyperpalatable food reward used in instrumental and Pavlovian training. O3—hyperpalatable food reward used only in Pavlovian training. O4—non-hyperpalatable food reward used only in Pavlovian training. 3-back—the percentage of correct trials of the 3-back working memory task. Go no go—the percentage of hit and correct rejection trials in the go no go task. Delay discounting—the k index derived from the delay discounting task. ^a^Indicates significant main effect of sleep condition; ^b^indicates significant main effect of satiation.

### Learning of response-outcome (R-O) knowledge

During the training phase (see Table 4 in “Methods” for detailed PIT procedures), one participant did not achieve R-O knowledge during the last block of instrumental training query trials in NSD, and five of them in TSD. The difference was not statistically significant (*X*^2^ = 0.83, *p* = 0.36). All participants achieved S-O knowledge during the last block of Pavlovian training query trials in both conditions.

During the testing phase, query trials were presented to check whether participants remembered the R-O knowledge. All participants in NSD showed correct memory of the R-O knowledge, and three (4%) in TSD did not. Hence, the data from these three participants were excluded from the analysis, leaving data of 75 participants for the analysis of PIT data. The difference in R-O knowledge between NSD and TSD was not statistically significant (*X*^*2*^ = 2.06, *p* = 0.36).

### Specific transfer—biased responding by cue

Specific transfer was measured as the biased responding by cues in the PIT paradigm (see Table 4 in “Methods”), specifically, the differences in responding in the presence of CS- (no cue), CS-same (cue predicting the reward associated with the key), CS-different (cue predicting reward associated with the other key), CS-other-reward (cue predicting food rewards not associated with any key), and CS-no-reward (cue predicting the absence of reward). A 3-way (sleep × cue × satiation) repeated-measures ANOVA with the percentage of instrumental responses for the hyperpalatable food as the dependent variable was conducted. Evaluations of the residual plots suggested that the multivariate normality assumption was met. Three multivariate outliers were identified by evaluating the Mahalanobis distance, the distance between a data point and the center of a distribution, indicating unusual deviation from a multivariate normal distribution. Further evaluations found that these outliers were valid data points and removing them did not make noticeable differences to the pattern and significance of the results. Hence, the analysis of the full dataset was conducted and reported here.

**Figure 1:**
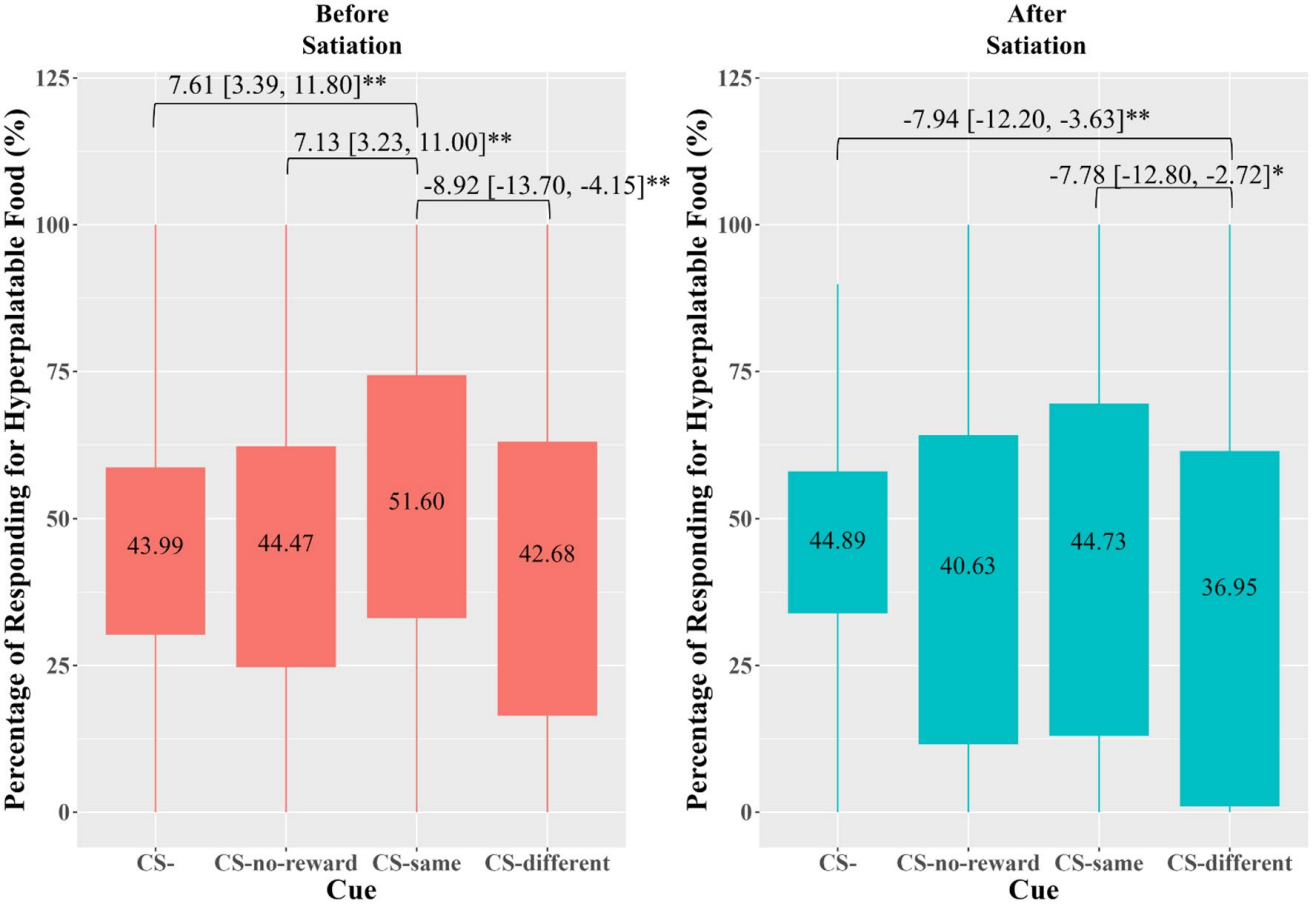
Percentage of Responding for the Signaled Hyperpalatable Food Before and After Satiation (Combined Sleep Conditions). *Note.* CS- = no cue. CS-same = cue predicting the reward associated with the key. CS-different = cue predicting reward associated with the other key. CS-no-reward = cue predicting the absence of reward. The box area covers the middle 50% of the data values. The upper whisker covers the top 25% of the data values and the lower whisker covers the bottom 25% of the data values. The values displayed in the boxes indicate the mean values. The satiation × cue 2-way interaction is significant, showing that the effect of cue is significant only before satiation. Significant post-hoc comparisons are indicated along with the mean difference and the 95% confidence interval. *Bonferroni-corrected *p* < 0.05; ** *p* < 0.01.

The sleep × cue × satiation 3-way interaction was non-significant but marginal (*F*[3,222] = 2.48, *p* = 0.062). The satiation × cue 2-way interaction was significant (*F*[3,222] = 2.75, *p* = 0.044). The simple main effect of cue was significant before satiation (*F*[2.68,199] = 6.48, *p* = < 0.001) and after satiation (*F*[2.58,191] = 5.71, *p* = 0.002) As shown in Fig. 1, participants preferred the non-hyperpalatable food when there was no cue (CS-)—less than 50% of responses were made to obtain the hyperpalatable food either before or after satiation. Before satiation, there was a significant elevation of biased responding for the hyperpalatable food in the presence of CS-same compared to CS- (Cohen’s *d *= 0.42), CS-no-reward (Cohen’s *d *= 0.42), and CS-different (Cohen’s *d *= 0.39), supporting the presence of a specific transfer effect. After satiation, biased responding for the hyperpalatable food in the presence of CS-same was not significantly different from CS-, indicating that the specific transfer effect was sensitive to satiation. Nonetheless, the biased responding in the presence of CS-different was significantly suppressed compared to CS- (Cohen’s *d *= − 0.41) and CS-same (Cohen’s *d *= 0.32), indicating some degree of the effect of cues on biased responding even after satiation.

### Specific transfer—rate of responding by cue

The rates of responding for the signaled food across different sleep × satiation conditions were further examined by two 3-way (sleep × cue × satiation) repeated-measures ANOVA, one with the rate of responding for the hyperpalatable food as the dependent variable, the other with the rate of responding for non-hyperpalatable food as the dependent variable. Evaluations of the residual plots suggested that the multivariate normality assumption was met with no extreme multivariate outliers. Hence, all data were analyzed.

**Figure 2:**
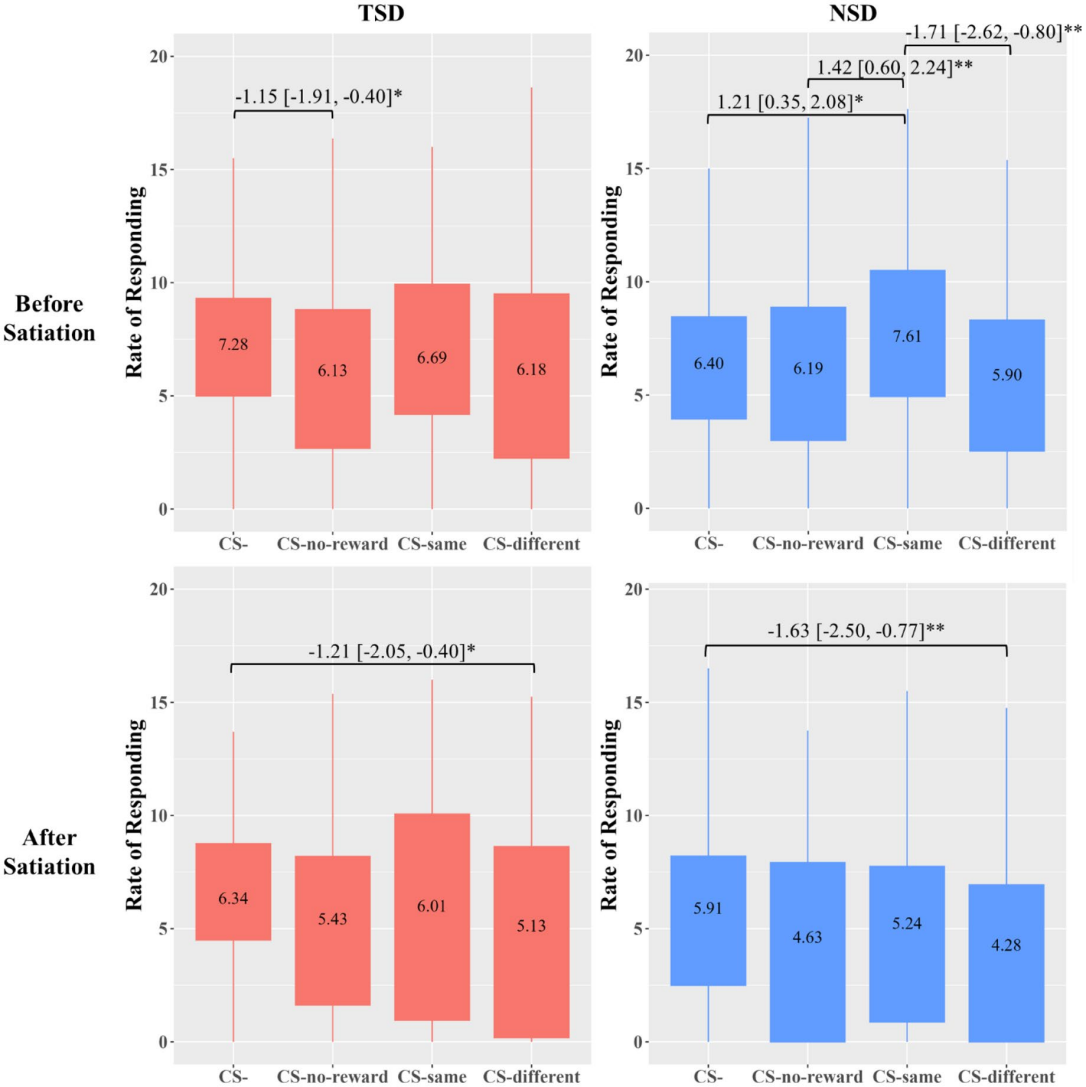
Rate of Responding for Hyperpalatable Food by Sleep and Satiation Conditions. *Note.* TSD, total sleep deprivation. NSD, normal sleep duration. The box area covers the middle 50% of the data values. CS- = no cue. CS-same = cue predicting the reward associated with the key. CS-different = cue predicting reward associated with the other key. CS-no-reward = cue predicting the absence of reward. The upper whisker covers the top 25% of the data values and the lower whisker covers the bottom 25% of the data values. The values displayed in the boxes indicate the mean values. The sleep × cue × satiation interaction effect is significant. Significant post-hoc comparisons are indicated along with the mean difference and the 95% confidence interval. *Bonferroni-corrected *p* < 0.05; ** *p* < 0.01.

The sleep × cue × satiation 3-way interaction on the rate of responding for the hyperpalatable food was significant (*F*[3,222] = 2.83, *p* = 0.039). The sleep × cue 2-way interaction effect was significant only before satiation (*F*[3,222] = 3.58, *p* = 0.015) but not after satiation (*F*[3,222] = 0.18, *p* = 0.909). Post-hoc comparisons showed that (see Fig. 2), before satiation and following NSD, the rate of responding for hyperpalatable food was significantly elevated in CS-same compared to CS- (Cohen’s *d* = 0.32), CS-no reward (Cohen’s *d* = 0.40), and CS-different (Cohen’s *d* = 0.43), indicating the presence of a specific transfer effect in NSD. Contrarily, this effect was not observed in TSD.

**Figure 3:**
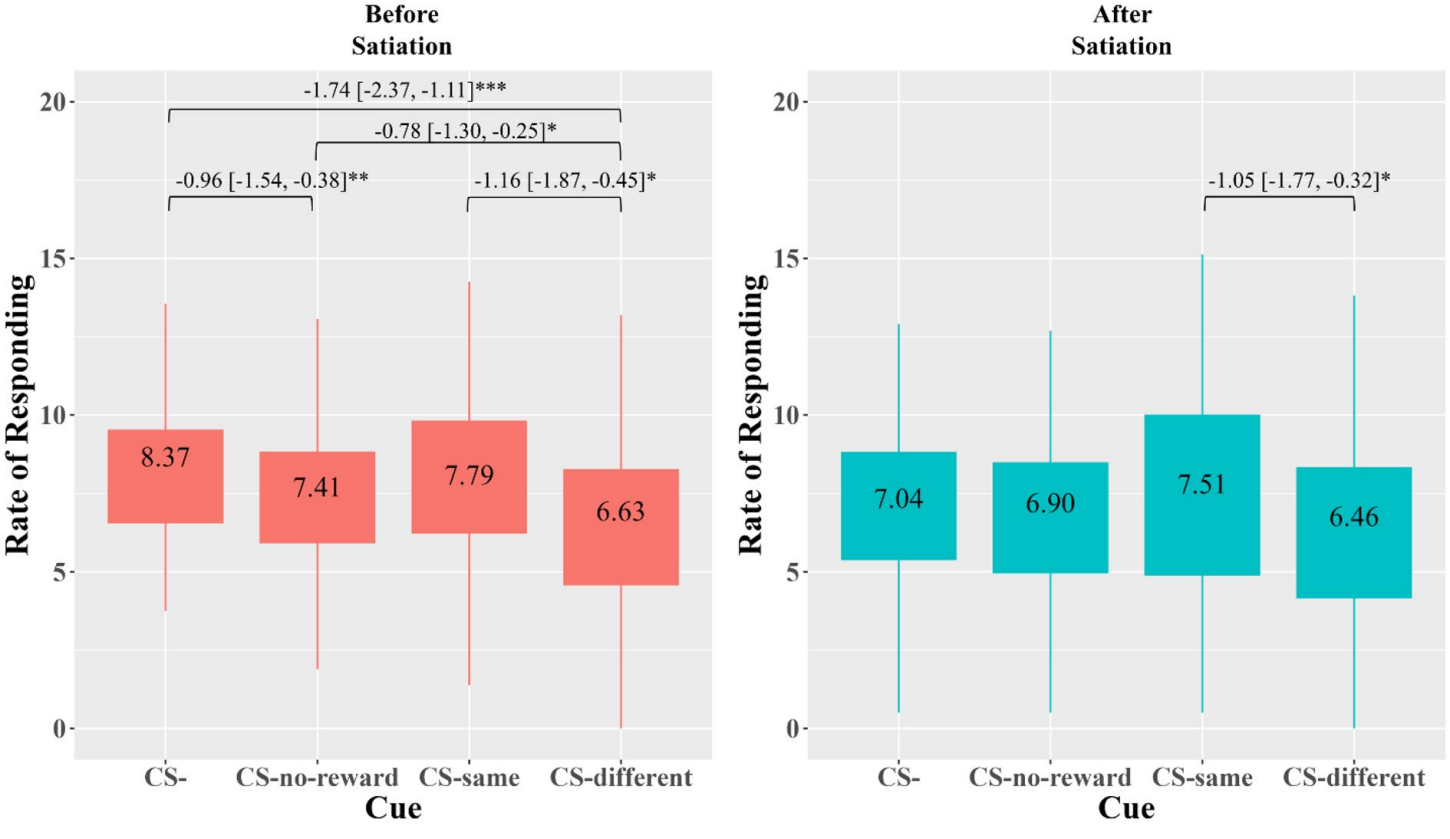
Cue × Satiation Interaction Effect on the Rate of Responding for Non-Hyperpalatable Food. *Note.* CS- = no cue. CS-same = cue predicting the reward associated with the key. CS-no-reward = cue predicting the absence of reward. CS-different = cue predicting reward associated with the other key. The box area covers the middle 50% of the data values. The upper whisker covers the top 25% of the data values and the lower whisker covers the bottom 25% of the data values. The values displayed in the boxes indicate the mean values. Significant post-hoc comparisons are indicated along with the mean difference and the 95% confidence interval. *Bonferroni-corrected *p* < 0.05; ** *p* < 0.01; *** *p* < 0.001.

On the other hand, the sleep × cue × satiation 3-way interaction on the rate of responding for the *non-hyperpalatable* food was non-significant, but marginal (*F*[3,222] = 2.49, *p* = 0.061). The cue × satiation 2-way interaction was significant (*F*[3,222] = 3.06, *p* = 0.029). The effect of cue was significant before (*F*[3,222] = 10.90, *p* < 0.001) and after satiation (*F*[2.71, 200] = 3.71, *p* = 0.015). As shown in Fig. 3, before satiation, there was not significant elevation in the rate of responding for the non-hyperpalatable food in the presence of CS-same, but suppressed rates of responding in the presence of CS-different compared to CS- (Cohen’s *d* = − 0.63), CS-same (Cohen’s *d* = − 0.38), and CS-no reward (Cohen’s *d* = − 0.34). After satiation, the rate of responding in the presence of CS-different was significantly lower than that in CS-same (Cohen’s *d* = − 0.33).

### General transfer

**Figure 4:**
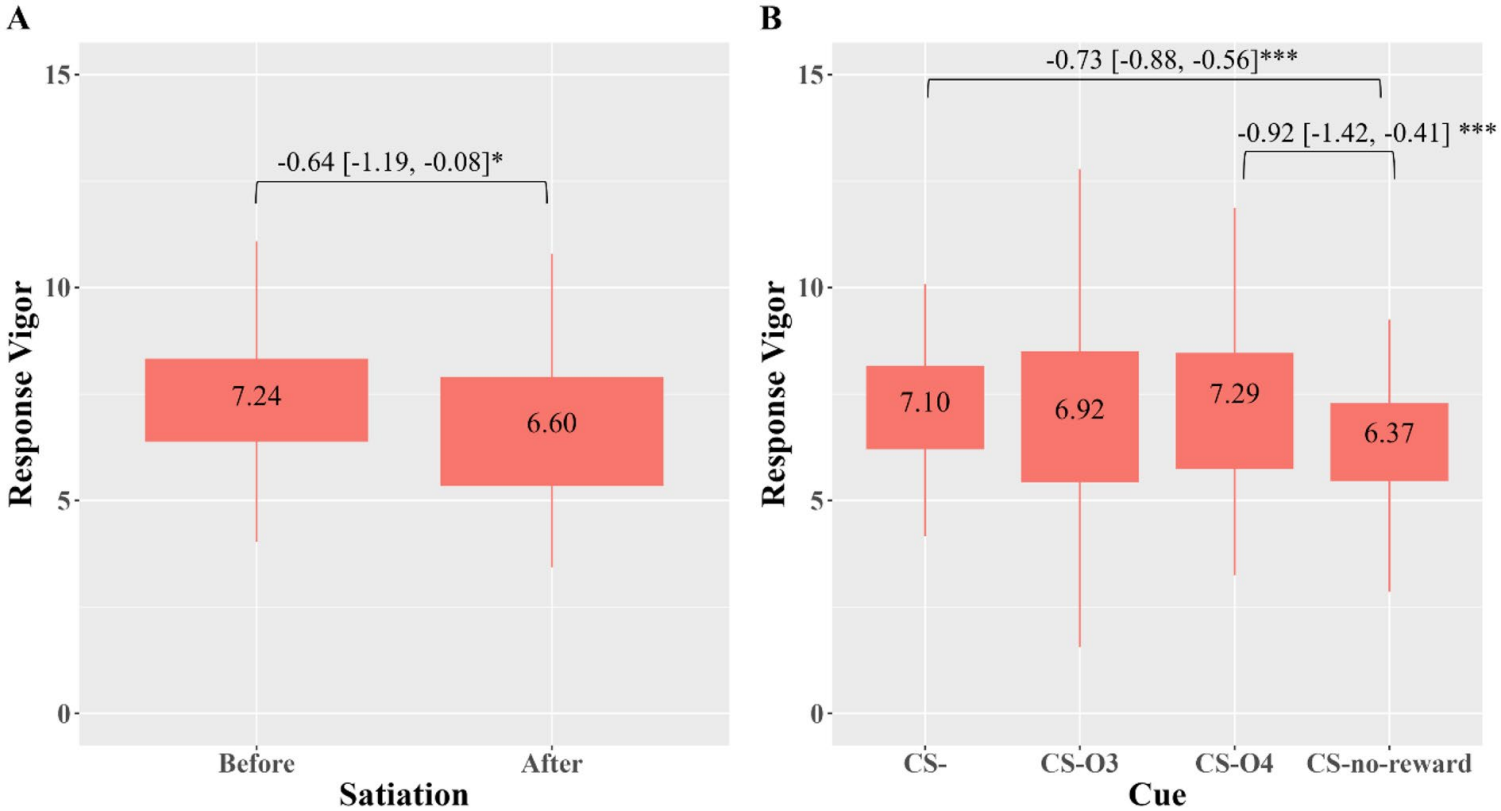
Main effects of satiation and cue on response vigor. *Note.* CS- = no cue. CS-O3 = cue predicting another reward (hyperpalatable). CS-O4 = cue predicting another reward (non-hyperpalatable). CS-no-reward = cue predicting the absence of reward. Panel A indicates the main effect of satiation. Panel B indicates the main effect of Cue. The box area covers the middle 50% of the data values. The upper whisker covers the top 25% of the data values and the lower whisker covers the bottom 25% of the data values. The values displayed in the boxes indicate the mean values. Significant post-hoc comparisons are indicated along with the mean difference and the 95% confidence interval. *Bonferroni-corrected *p* < 0.05; *** *p* < 0.001.

*General transfer* was measured by the number of all key presses in the presence of CS-other reward (C3 and C4) compared to CS- and CS-no-reward. A 3-way (sleep × cue × satiation) repeated-measures ANOVA was conducted with the combined response vigor as the dependent variable. Evaluations of the residual plots suggested that the multivariate normality assumption was met with no extreme multivariate outliers. Hence, all data were analyzed. All interaction effects were non-significant, but the main effects of satiation (*F*[1,74] = 23.34, *p* < 0.001) and cue were significant (*F*[1,74] = 71.47, *p* < 0.001). As expected, response vigor was significantly suppressed after satiation (see Fig. 4A; Cohen’s *d* = − 0.26). Response vigor in the presence of CS-no-reward was significantly suppressed compared to CS- (Cohen’s *d* = − 1.04) and C4 (see Fig. 4B; Cohen’s *d* = − 0.42).

### Correlations between transfer effects and covariates

Both within- and between-individual correlations of specific transfer and general transfer effects with ratings of hunger, stress, liking of each of the food items, caloric consumption, go no go task, working memory, and delay discounting were examined. None of the correlations were significant after Bonferroni adjustments of *p*-values (see Table 3).

**Table 3:** Within- and between-individual correlations of specific transfer and general transfer effects with other variables.

Variables	Within		Between	
	Specific transfer effects	General transfer effects	Specific transfer effects	General transfer effects
O1 liking	0.01	− 0.11	0.12	0.16
O2 liking	− 0.03	0.11	0.08	− 0.09
O3 liking	− 0.003	0.02	− 0.01	0.09
O4 liking	0.08	− 0.05	− 0.06	0.01
Hunger	0.10	− 0.06	− 0.02	− 0.30
Stress	0.01	− 0.02	0.14	0.13
3-back (%)	0.07	− 0.04	− 0.08	− 0.07
Go no go	0.11	0.02	− 0.03	0.06
Delay discounting	− 0.04	0.04	0.15	0.15
Calorie consumed (kcal)	− 0.16	0.11	− 0.02	− 0.06

*Note.* O1—high-palatability food reward used in instrumental and Pavlovian training. O2—low-palatability food reward used in instrumental and Pavlovian training. O3—high-palatability food reward used only in Pavlovian training. O4—low-palatability food reward used only in Pavlovian training.

## Discussion

The present study examined the effects of one-night TSD and satiation on food-related Pavlovian-instrumental transfer effects using a within-individual randomized, crossover experimental design. Cue-elicited food-seeking responding was found to differ in different combinations of sleep and satiation conditions. This finding was novel. However, contrary to the hypothesis, specific transfer effects were found in NSD before satiation but not in TSD. Sleep by satiation interactions were not found for the general transfer effects.

The present findings did not support the hypothesis that one-night TSD would exacerbate habitual eating and lead to greater cue-elicited motivation for eating even after satiation. Rather, these findings appear to align with the literature on sleep and appetitive conditioning in animal samples. In a systematic review of animal studies, disruption of sleep following learning was found to impair the acquisition, consolidation, and extinction of appetitive behaviors when natural reinforcers such as food were employed^41^. And, this alteration was unlikely attributable to learning or memory deficits. As shown in a study of sleep deprivation in rats, sleep-deprived rats were found to achieve similar probabilities in performing a behavioral response to a rewarded outcome as well-rested rates, indicating similar degrees of learning/memory; however, only sleep-deprived rats exhibited decreased responding over time, suggesting an alteration in motivational processes^42^.

Chen et al.^34^ was the only human study of the effect of sleep deprivation on appetitive conditioning, and although they found that one-night TSD increased biased responding for a devalued outcome using the slips-of-the-action task, they did not control for the impact of TSD on information encoding as learning was conducted after TSD. Indeed, in their Experiment 2 when learning occurred before sleep deprivation they did not find such an effect. Consistently, the present study found that sleep deprivation did not increase cue-elicited appetitive behavior. It should be noted that the research on the effects of sleep on reward conditioning in human is scarce. It is recognized that reward conditioning processes and habitual vs goal-directed processes are frequently regarded as a proposed mechanism underpinning appetite disorders in humans^43^. The present findings highlighted the role of sleep disruption in appetitive conditioning, but given the unexpected results, future research is needed to further disentangle the effects of sleep disruption on these processes.

Food-related specific transfer effects were quite consistently observed in previous studies^38,39^. Similarly, a specific transfer effect was found in NSD before satiation in the present study. The percentage of responding for the signaled food increased from 42% in CS- to 56% in CS-same and the specific transfer effect disappeared after satiation. A larger specific transfer effect was reported by Watson et al.^39^, who found greater than 30% increases in biased responding for the signaled food before satiation and this effect remained significant even after satiation. While the instrumental and Pavlovian training procedures in Watson et al.^39^ and the present study were close to identical, the order of the non-cued and cued testing trials in Watson et al., was counterbalanced, with a break in between. In the present study, the non-cued trials were presented first, followed by the cued trials without a break. As the transfer tests were conducted in extinction with no rewards given immediately, the first-presented set of non-cued tests could have decreased the strengths of the R-O associations and led to decreased responding in the subsequent cued trials. Without a break, there could also be a fatigue effect on responding in the cued trials. This methodological artifact could have masked the specific transfer effects to some degree. Future studies presenting a randomized order of non-cued and cued trials could eliminate the order effect and better evaluate whether food-related specific transfer effects would be sensitive to satiation in different sleep conditions.

Although the PIT effects are conceptualized as a reflection of habitual control of food-seeking behavior in the present study, other theoretical accounts of the PIT effects have been proposed arguing that the PIT effects are goal-directed rather than habitually-controlled^44^. Recent theoretical developments also argue that overeating and other suboptimal behavior are goal-directed rather than habitually controlled^45,46^. Indeed, we found that the specific transfer effect observed in NSD was sensitive to outcome devaluation, consistent with the goal-directed account. The specific transfer effect being sensitive to outcome devaluation was also consistent with previous failed attempts to experimentally induce outcome-insensitive instrumental behavior^47^. These findings might suggest that future studies on the effect of sleep deprivation on the motivational aspect of eating behavior may not have to be theoretically limited to either the habit or goal-directed account, but to focus on the dissociable processes underlying cue reactivity and insensitivity to outcome devaluation. For instance, the double dissociation account of the PIT effects, i.e., conditioned cues and outcome devaluation could independently influence instrumental actions^48^, maps well onto the distinction between cue-elicited eating, which is normative, and eating despite satiation, which is more closely related to disordered eating and obesity. The use of the PIT paradigm allows for the examination of these two processes independently and interactively. The present study is the only study, to the best knowledge of the author, that has examined the effect of sleep deprivation on food-related PIT effects in human using a well-controlled within-individual experimental design.

Given the aforementioned order effect, the findings on the general transfer effect should be interpreted with caution. The absence of any interaction effects with sleep or satiation suggested that sleep conditions did not have any influence on the general transfer effect. The main effect of cue showed that the response vigor in the presence of CS-no reward was suppressed compared to CS-O4. It was a possibility that if the order effect was not present, an elevation of response vigor compared to that in CS- might be observed in the presence of CS-O4.

In the exploratory correlation analysis, specific transfer effects were not correlated with hunger, stress, liking of the food rewards, caloric consumption, response disinhibition, working memory, and delay discounting, suggesting that specific transfer was independent from homeostatic eating and hedonic eating, and was not affected by state levels of stress, response biases, or executive functioning. Quail et al.^38^ found that trait levels of stress measured by the DASS were correlated with transfer effects. Although the DASS was employed in the present study, we screened out individuals with higher scores on the DASS and thus resulted in a restricted range of scores in the sample. Hence, correlations with DASS were not performed.

It should be noted that caloric consumption during satiation did not differ between TSD and NSD. Although this finding contradicted those from some previous studies showing that sleep deprivation led to increased caloric intake^15^, it was in line with other studies in which total caloric intake per day was not found to differ following sleep deprivation^49^. In particular, a recent study using a 5-night 6.5-hour partial sleep deprivation experimental design found a non-significant difference in total caloric consumption per day between the sleep deprivation and normal sleep duration conditions^19^. Duraccio et al.^19^ found an increase in caloric consumption only during late evening, suggesting that the effects of sleep duration on caloric consumption might be modulated by the timing of food consumption. Similarly, a previous naturalistic, observational study found that habitual short sleep duration was associated with decreased consumption of calorie-dense food in breakfast^50^. The fact that the PIT and satiation procedures were conducted in the morning in the present study might explain why caloric consumption was not found to differ following sleep deprivation. The absence of a difference in caloric consumption in the present study also highlighted the complexity of the relationship between sleep deprivation and eating—timing of food consumption, food cues and availability, and food palatability, could modulate eating behavior following sleep deprivation. Examining sleep and caloric consumption alone may not capture important changes in the mechanisms underlying eating behaviors, such as cue-elicited mechanisms as shown in the present study.

The findings of this study should be interpreted considering the following. First, the current sample was overrepresented by young, healthy, normal-weight individuals who were likely not at risk of obesity and obesogenic eating behavior, the present findings may not be generalizable to less-healthy populations, who might be particularly susceptible to the effects of sleep deprivation. Moreover, 25 out of the 121 initially recruited participants dropped out from the experiment. Many of them withdrew from the study because they could not follow through with the normal sleep requirement during the washout period. Although these participants did not differ significantly on the demographic and clinical variables from those who completed the experiment, they could differ on other unmeasured variables. Second, the NSD condition was conducted in participants’ homes. Although sleep duration was validated by actigraphy, the differences in sleep environments across individuals and the commute to the laboratory in the morning to participate in the PIT tests could have introduced noise to the results. Third, the effect of satiation was tested with a within-individual design, meaning that the results of the post-satiation PIT test may be confounded by the effect of extinction that occurred during the first, pre-saturation PIT test. Similarly, the order effect of the non-cued and cued tests introduced noise to the interpretation of the specific and general transfer effects. Fourth, one-night TSD may not be representative of real-life patterns of chronic partial sleep deprivation. Future studies employing a multi-day partial sleep deprivation design will have greater ecological validities. Finally, measures of alertness and sleepiness were not obtained which would have allowed further evaluations of the relationship between these sleep-related constructs and transfer effects. Measures of the desire to eat the food rewards were not obtained either which was distinct from food liking. They could be compared to the findings of PIT effects to shed light on the convergence between subjective food-seeking motivation and PIT effects.

Despite the aforementioned limitations, this study constituted the first empirical test of the effects of one-night TSD on food-related PIT effects. Although the findings did not support the hypothesis, they suggested that sleep deprivation could alter the PIT effects. Future studies are needed to further examine the influence of sleep parameters on motivational processes underlying eating.

## Methods

### Transparency and openness

The study design, hypotheses were pre-registered with the Open Science Foundation prior to data collection (https://osf.io/rk35y/?view_only=838f8c3125af4a2fac5600895775f21c). The analysis plan was not pre-registered. The de-identified data and analytic codes were uploaded to an open repository^51^. All research materials are available for access upon request for research and review purposes. An ethical approval was obtained from the University Human Ethics Committee (Ref# EA1905006) prior to participant recruitment, and all recruited participants provided electronic informed consent prior to the commencement of the study procedures. Participants were compensated $50USD for participating in the experiment.

### Participants

Generally healthy adults (aged 18–65) who were free of sleep disorders, psychiatric disorders, eating disorders, and mood disorders and had normal habitual sleep patterns (7–9 h of sleep per night from 22:00 to 08:00) were recruited to the study to minimize any confounding impact of mental or physical illness on eating and to ensure that the participants could follow the experimental sleep manipulation procedures. Specific inclusion criteria were (a) aged 18–65 years and (b) having habitual sleep of seven to nine hours per night between 22:00 and 08:00, confirmed by seven days of actigraphy. Exclusion criteria were (a) having elevated sleep disturbances, indicated by a score of 7 or above on the Chinese version of the Pittsburgh Sleep Quality Index (PSQI)^52,53^; (b) having evening chronotypes indicated by a score < 42 on the morningness–eveningness questionnaire (MEQ)^54^; (c) having any self-reported current or history of psychiatric disorders including eating disorders, obsessive-compulsive disorders, substance abuse, bipolar disorder, or severe mental illnesses; (d) having any eating disorder symptoms indicated by a score > 16.5 on the Eating Disorders Diagnostic Scale (EDDS)^55^; (e) having severe depressive or anxiety symptoms indicated by a score > 12 on the depressive subscale or a score > 8 on the anxiety subscale of the Depressive Anxiety and Stress Scales (DASS)^56^; (f) having any medical conditions that could be worsened by one-night TSD; g) currently taking any psychiatric medications; (h) currently trying to lose weight; and, (i) having allergies or aversion to the experimental food stimuli. Additionally, women who scored > 30 on the shortened premenstrual assessment scale^57^, indicative of premenstrual syndrome, were scheduled for the study during the follicular phase of their menstrual cycles to minimize the confounding effects of hormonal changes on appetite^58^.

### Procedures

#### Screening and confirmation of habitual sleep duration

Participants completed an online survey consisting of the screening measures. Those who met the initial inclusion criteria were provided with an actigraph (ActiGraphTM model GT9X Link) to confirm their habitual sleep patterns. Those who met all the inclusion and exclusion criteria were invited to enroll in the experiment.

#### Experimental sleep manipulation

**Figure 5:**
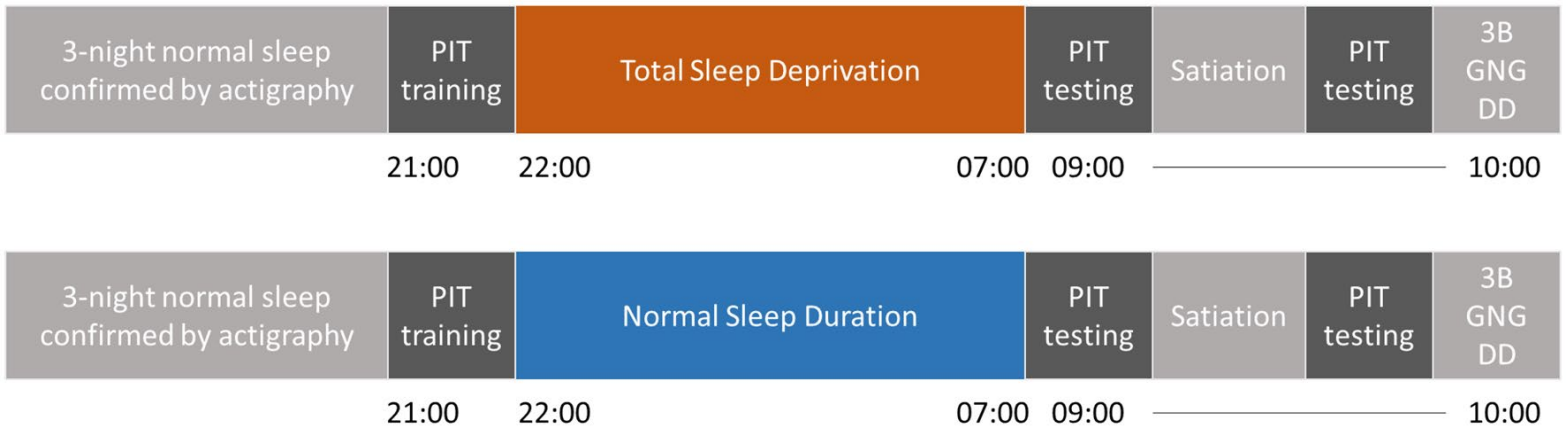
Experimental Procedures. *Note.* PIT—Pavlovian-instrumental transfer paradigm. 3B—3-back working memory test. GNG—Go no go test. DD—delay discounting task. The sequence of the two conditions was randomized.

A within-individual randomized crossover design with a minimum three-day washout period was employed (see Fig. 5). Participants were randomized to begin with either the TSD or the normal sleep duration (NSD) condition, followed by at least 3 days of washout prior to the other sleep condition. During TSD, participants were settled in an approximately 350 square-feet room decorated like a living room and ensured to be awake until the next morning by research assistants. They could use their laptops and engage in any activity that did not involve vigorous physical activity. Participants were not allowed to consume any alcohol, nicotine-containing substances, caffeine, or to nap during the study procedures, although they could eat self-prepared snacks in the laboratory until 04:00. The NSD condition was conducted in the participants’ homes. Participants were instructed to sleep for seven to nine hours between 22:00 and 07:00, with the duration of sleep confirmed by actigraphy. All participants wore an actigraph throughout the study to cross-validate the sleep manipulation, especially during the washout period and in the NSD condition.

#### Pavlovian-instrumental transfer (PIT) paradigm

As shown in Fig. 5, the PIT employed in the present study consisted of the instrumental and Pavlovian training phases and the two testing phases with satiation in between. The PIT training phases were completed prior to sleep manipulation to ensure that any differences in the PIT tests were not due to inadequate or unsuccessful learning associated with sleep loss. The PIT testing phases were completed in the following morning at the same time in both conditions to control for circadian influences. Participants also completed ratings of hunger, stress, and food liking at the beginning of the PIT testing phase, and completed tests of working memory, response inhibition, and delay discounting, at the end of the PIT testing phase. The PIT paradigm was programmed using the PsychoPy v3.2 software package and administered to each participant individually on a desktop computer with a 24-inch monitor while the participant sat in a quiet room. Participants were told that they would be presented with food pictures and actual food rewards and should follow the on-screen instructions to obtain the food rewards (instructions included in Supplementary 1). They were asked to not consume any food within four hours prior to the PIT training and testing phases. Participants were instructed to use their dominant hand to press the keys and pick up the food items throughout the PIT. The food items were placed to the side of the computer monitor corresponding to the participants’ dominant hand.

The classic PIT paradigm is widely used in animal and human studies to model the influence of Pavlovian cues, i.e., conditioned cues that become associated with an outcome via classical conditioning (stimulus-outcome [S-O] association), on instrumental responses learned via operant conditioning (response-outcome [R-O] association)^37^. The transfer effects derived from the PIT paradigm refer to the phenomenon that the presence of Pavlovian cues enhances the probability and/or the vigor of instrumental responses. There are two types of transfer effects. The *specific transfer effect* refers to the increase in the probability and/or the frequency of performing an instrumental response by the presence of the conditioned cue signaling the outcome associated with the instrumental response. The *general transfer effect* refers to the increase in the vigor of an instrumental response by the presence of cues signaling an outcome that is not associated with the instrumental response but generates a similar motivational state.

The procedures of the PIT paradigm used in the present study is presented in Table 4. The number of key presses (R1 and R2) in response to the presence of CS- (no cue), CS-same (cue predicting the reward associated with the key), CS-different (cue predicting reward associated with the other key), CS-other-reward (cue predicting food rewards not associated with any key), and CS-no-reward (cue predicting the absence of reward) were recorded. There were 10 non-cued trials (CS-) and 50 cued trials (10 presentations of each of the five Pavlovian cues (C1-C5). The 10 non-cued trials were presented first, followed by 50 cued trials presented in a random order. *Specific transfer* was measured by biased responding in the presence of CS-same compared to CS-no-reward, i.e., the percentage of R1 or the rate of R1 responses in the presence of C1 (conditioned cue associated with O1) compared to C5 (control cue associated with no reward) for high-palatability food and the percentage of R2 or the rate of R2 in the presence of C3 (conditioned cue associated with O2) compared to C5 for low-palatability food. *General transfer* was measured by the number of key presses (combined R1 and R2) in the presence of C3 and C4 (CS-other-reward) compared to CS- and CS-no-reward.

**Table 4:** Procedures of the Pavlovian-instrumental transfer (PIT) paradigm.

Phase	Goal	Procedures & visual illustration
Instrumental training phase	Participants acquire the associations between two responses, pressing X (R1) and pressing M (R2) and two corresponding food rewards. One food reward is considered high-palatability (O1), the other low-palatability (O2). The key-palatability associations were counterbalanced across participants	Participants were asked to press either the X or M key as many times as they wanted in response to the appearance of a white box on the screen until an image of a food outcome appeared on the screen. They were told that each of the two keys was associated with a food reward and that they should learn the associations between the two keys and the two corresponding food rewards. Each participant was presented with four blocks of trials in which the two food rewards were both available three times, in random order (24 trials in total). A variable ratio schedule of 10 was used, i.e., after 5–15 presses of a key, an image of the associated food reward appeared on the screen for 1 s. After every fourth appearance of the image of a food reward, the participant was prompted to consume a bite of the food reward and was given 10 s to do so. At the end of the second and fourth instrumental blocks, a block of four instrumental query trials will be run to test the participants’ ability to learn the associations. In each instrumental query trial, the participant was presented with a picture of either O1 or O2 and was asked to press the X or M key to indicate which key was associated with O1 and O2. They received immediate feedback on their response, either “correct” or “incorrect”.
Pavlovian training phase	Participants acquire the associations between five graphic patterns (C1-C5) and five outcomes (O1-O5). O1 and O2 are the same as in the instrumental training phase; O3 and O4 are two other food rewards, one is high-palatability, the other low-palatability; O5 is the absence of any reward (control)	Participants were asked to learn the associations between the graphic patterns and the outcomes. The graphic cue appeared on the screen for 2 s and was then overlaid with the picture of the associated food reward or no reward for 1 s. Every fourth time that a specific food reward picture was presented, the participant was prompted to consume that food. Query trials were used to ensure successful learning. The Pavlovian training phase consisted of four blocks, during each of which the five cues will be presented twice in random order (40 trials in total). At the end of the second and fourth Pavlovian blocks, a block of five Pavlovian query trials will be run to test the participants’ ability to learn the associations. In each Pavlovian query trial, the participant was presented with a graphic pattern and a food reward and was asked to indicate if they were associated. They received immediate feedback on their response, either “correct” or “incorrect”.
Testing phase	Evaluate specific and general transfer effects	Participants were presented with two instrumental query trials to check if they remembered the associations between the keys and food outcomes and two demo trials to ensure that they understood the instruction for the testing phase. They were told that they could press either X or M as many times as they wanted when they see a white box on the screen to earn the food rewards based on the associations they learned in the previous night. However, they would not receive any feedback or rewards after each trial but would be given the food rewards after completing the testing phase. In the non-cued tests, the white box was presented against a blank background. In the non-cued tests, the graphic cues were presented overlaying the white box for 3 s. There were 10 non-cued trials and 50 cued trials (10 presentations of each of five the Pavlovian cues, in random order (50 trials in total). The 10 non-cued trials were presented first, followed by 50 cued trials presented in a random order.

*Note.* Specific and general transfer effects were derived from the PIT paradigm. Specific transfer was measured by the percentage of R1 in the presence of C1 (conditioned cue associated with O1) compared to C5 (control cue) for high-palatability food and the percentage of R2 in the presence of C3 (conditioned cue associated with O2) compared to C5 for low-palatability food. General transfer was measured by the number of key presses (combined R1 and R2) in the presence of C3 and C4 compared to CS- and CS-no-reward.

The PIT paradigm used in the present study was largely similar to that in Watson et al.^39^ and Quail et al.^38^ with two exceptions. In Watson et al.^39^, there was only one CS-other-reward, which was associated with a non-hyperpalatable food reward. In the present study, there were two CS-other-reward, one hyperpalatable and one non-hyperpalatable, to control for food palatability. Because hyperpalatable food is suggested to have properties similar to addictive drugs and might induce greater motivational changes^59^, food palatability was counterbalanced in the instrumental and Pavlovian training phases. And hence, there were a total of four food rewards, two hyperpalatable (O1 and O3) and two non-hyperpalatable (O2 and O4) in the present study. Additionally, both food rewards were satiated during outcome devaluation. Rather than satiating on one food reward and examining the biased selective responding for one food reward, both food rewards were satiated during outcome devaluation and the rate of responding for the hyperpalatable food and that for the non-hyperpalatable food were examined to evaluate transfer effects associated with the two foods with different palatability.

#### Satiation

Satiation was conducted to devalue the food rewards. Participants completed the PIT testing phase twice, once before and once after satiation. During satiation, each participant was given an excess of the two food rewards used in the instrumental training phase to consume in the next 20 min. They were told they could eat as much as they wanted and that they could use their phones during the task. Caloric consumption was recorded in each sleep condition.

#### Selection of food rewards

Two hyperpalatable foods and two low-palatability foods were chosen for each participant after controlling for their subjective liking of the food. The definition of hyperpalatable food was adopted from Fazzino et al.^60^, characterized by food composed of high (1) fat and sodium, (2) fat and sugar, or (3) carbohydrates and sodium. During screening, participants were asked to rate their liking of 18 food items (9 hyperpalatable, 9 non-hyperpalatable) on a 10-point Likert scale (1 = dislike it; 10 = love it). The two hyperpalatable and two non-hyperpalatable food items that received similar liking ratings (with a score of at least 3, indicating “don’t mind eating it”) for a participant were then used as the food rewards for that participant, to control for the impact of liking of the food on the PIT performance. Because the study adopted a within-subject design, each participant had to complete the PIT twice. Hence, two sets of food rewards were used to prevent carryover effects.

### Measures

#### Screening measures

##### DASS

The depression and anxiety subscale of the DASS-21 was used in the present study for screening^56^. The validated Chinese version of the DASS-21 was used^61^. The depression and anxiety subscales each consisted of seven items assessing depressive and anxiety symptoms on a 4-point scale from 0 to 3; higher scores indicated greater severity of symptoms. Those who scored above mild levels of depressive (> 13) and anxiety symptoms (> 9) were excluded from the study. The DASS-21 had excellent internal consistency in the screening sample (Cronbach’s α = 0.93).

##### EDDS

The EDDS was used to screen out individuals with significant levels of eating disorders symptoms^55^. The EDDS-DSM-5 version was used, and it was translated from English to Chinese using the forward and backward translation method. It consisted of 23 items assessing cognitive and behavioral symptoms of BED, BN, and AN. The composite score > 16.5, computed from summing items 1–17, was a validated cutoff for indicating the presence of disordered eating psychopathology^55^. Hence, those who scored 16.5 or above were excluded from the study. The EDDS had acceptable internal consistency in the screening sample (Cronbach’s α = 0.75).

##### MEQ

The MEQ^54^ was used to screen out participants who had evening chronotypes and could have difficulty following through with the experimental sleep manipulation procedures. It was translated from English to Chinese using the forward and backward translation method. Based on the overall score, respondents were categorized into five chronotypes: definite evening (scored 16–30), moderate evening (31–41), intermediate (42–58), moderate morning (59–69), and definite morning (70–86). Participants who were classified as having either definite evening or moderate evening chronotypes were excluded from the study. The MEQ had good internal consistency in the screening sample (Cronbach’s α = 0.82).

##### PAF

The shortened PAF consisted of 10 items assessing premenstrual symptoms of female participants’ last menstruation cycle^57^. The total score indicated different levels of symptom severity: absent (10), mild (11–29), moderate (30–44), and severe/extreme (45–60). Participants who scored above 30 were scheduled for the study during the follicular phase of their menstrual cycles to minimize the confounding effects of hormonal changes on appetite^58^. The PAF had good internal consistency in the screening sample (Cronbach’s α = 0.88).

##### PSQI

The PSQI, a widely used self-report measure of sleep quality consisting of 19 questions, was used to screen out individuals who had sleep disturbances. Seven component scores were derived from the PSQI which formed an overall sleep quality index in the range of 0–21; higher scores indicate worse sleep quality^52^. The validated Chinese version of the PSQI was used and the cutoff score of 7 was used to screen out individuals with potential sleep disorders^53^. The PSQI had acceptable internal consistency in the screening sample (Cronbach’s α = 0.65).

#### Measures of covariates

##### Rating of hunger, stress, and food liking

Participants rated their hunger and stress on visual analog scales (0–100) at the beginning of the PIT testing phases. They then reported their subjective liking of the food items on offer on visual analog scales (0–100) at the end of the PIT.

##### Working memory

The 3-back task^62^ was used to assess working memory. Participants were instructed to monitor a series of letters displayed in the center of the computer screen. They were told to press the y key when the letter presented was the same as the one displayed three trials prior and to press the u key when it was not the same. Participants were provided with feedback to indicate the correctness of their response: the letter turned green when the response was correct and red when it was incorrect. Participants completed two blocks of 20 trials. The percentage of correct trials was used as an index of working memory.

##### Response disinhibition

The go-no-go task^63^ was used to assess motor disinhibition. Participants learned which numbers were a “go” signal and which were a “no go” from feedback. Four blocks of 25 trials (5 go trials) were conducted. The percentage of trials with hit and correct rejection responses was used as an index of inhibition.

##### Delay discounting

The delay discounting task with hypothetical monetary rewards and the standard double-limit algorithm was used^64^. The discounting rate, *k*, was calculated and used as an index of a preference for immediate vs. remote rewards.

### Data analysis

#### Overview

The study’s primary hypothesis involved multiple within-subject factors with multiple levels, including sleep, cues, and satiation. Hence, repeated-measures ANOVAs were used to examine the primary hypotheses. Because interactions were involved, type III sum of squares were used. Examination of multivariate outliers, normality, and sphericity were conducted to evaluate if the data met the assumptions required for repeated-measures ANOVAs and if statistical adjustments were needed. The Greenhouse-Geisser correction was applied when the sphericity assumption was violated. Bonferroni-adjusted p-values were reported for post-hoc comparisons. All analyses were conducted using RStudio version 2023.03.1.

#### Specific transfer effects

The effects of sleep and satiation on specific transfer effects were first examined using a 3-way (sleep × cue × satiation) repeated-measures ANOVA with the percentage of instrumental responses for the hyperpalatable food as the dependent variable. Sleep had two levels: TSD vs NSD. Cue had four levels: CS-, CS-no-reward, CS-same, and CS-different. Satiation had two levels: before vs after satiation. Given that an increased percentage of instrumental responses could be attributable to suppressed responding in the presence of the cue predicting no reward rather than elevated responding in the presence of the cue predicting specific food reward, two additional 3-way (sleep × cue × satiation) repeated-measures ANOVAs were conducted, one with the rate of responding for the hyperpalatable food as the dependent variable, and the other with the rate of responding for the non-hyperpalatable food as the dependent variable.

#### General transfer effects

The general transfer effect was determined as the change in response vigor, computed by summing the number of both key presses by trial, in the presence of CS-other-reward compared to CS- and CS-no-reward. The effects of sleep and satiation on the general transfer effect were examined using a 3-way (sleep × cue × satiation) repeated-measures ANOVA.

#### Exploratory correlation analysis

To explore whether transfer effects were related to hunger, stress, liking of each food reward, response disinhibition, working memory, delay discounting, and caloric consumption, their correlations with transfer effects were examined. An index of specific transfer effect was formed by subtracting the rate of responding in the presence of CS-same by that in the presence of CS-. An index of general transfer effect was formed by subtracting the combined response vigor in the presence of CS-other-reward compared to CS-. Because participants completed these measures multiple times, both within-individual correlations and between-individual correlations were examined. The Bonferroni-adjusted significance level was used (0.05/10 = 0.005).

#### Sample size calculation

A medium effect size (partial eta-square = 0.07) was observed for the effect of one-night TSD on behavioral responsiveness to the devalued outcome in Chen et al.^34^. Although a different test, i.e., the PIT, was used in the present study, a medium effect size was the closest estimate of the expected effect size. Hence, a priori power analysis was conducted to estimate the minimum N needed for a medium effect for the primary hypothesis (partial η2 = 06). Given that multiple within-individual factors were involved in the analysis, the power analysis was conducted using the most conservative tests for complex within-individual factors, repeated-measures MANOVA. Using G*Power for a within-subject factor in repeated-measures MANOVA, N = 62 is required for β = 0.90 with α = 0.05 and ρ = 0.3. Considering that some participants might not follow through with all the experimental procedures or successfully learn the S-O and R-O associations in the PIT paradigm, the target N was set at 100.

### Supplementary Information

Supplementary Information.

## Data Availability

Data and analysis codes are available on 10.25442/hku.21904359.
