# Supplementary material for: Effects of sleep deprivation on food-related Pavlovian-instrumental transfer: a randomized crossover experiment
Source: Sci Rep. 2024 May 1;14:10029. doi: 10.1038/s41598-024-60223-2 (PMC11063131; doi:10.1038/s41598-024-60223-2)
Supplement: Supplementary file 1 — Supplementary Information. [file 41598_2024_60223_MOESM1_ESM.docx]

Instructions for the Pavlovian-instrumental transfer paradigm

Instrumental training phase:

In the following task, you are required to learn the association between key presses and food rewards. In each trial, a white box will appear in the middle of a black screen on the computer. The white box contains one of the two food rewards. When you see the white box, you will press either the X or M key using your index finger of your dominant hand. When you press a sufficient number of the correct key, you will be able to see the image of the food reward inside the white box. The number of presses required for the food image to show varies in each trial. After you see a food image for 4 times, you will be given the food for taste as a reward. At the end, you will be tested on whether you know which key is associated with which food.

Pavlovian training phase

In the following task, you will see a white box in the middle of the screen overlaid with a graphical pattern on the screen. In some trials, you will see a food item inside the white box. Your goal is to learn the association between graphical patterns and foods. In this task, you will not need to press any key, but you will have to pay attention to the screen. At the end, you will be tested on whether you know which pattern is associated with which food rewards.

Testing phase:

In the following task, you will see a white box and some graphic patterns on the screen. Whenever you see the white box, you can start pressing either X or M key to earn food rewards. Based on your learning from a previous task you performed last night, X and M keys are associated with specific food rewards. You should ignore the graphical patterns you see and press the key that is associated with the food reward you prefer, for as many times as you want. You will not see any food images in this task nor will you be given any food during this task. You will receive the food rewards after you complete this task.
